# Structural, Functional, and Metabolic Alterations in Human Cerebrovascular Endothelial Cells during *Toxoplasma gondii* Infection and Amelioration by Verapamil In Vitro

**DOI:** 10.3390/microorganisms8091386

**Published:** 2020-09-10

**Authors:** Alaa T. Al-sandaqchi, Victoria Marsh, Huw E. L. Williams, Carl W. Stevenson, Hany M. Elsheikha

**Affiliations:** 1 Faculty of Medicine and Health Sciences, School of Veterinary Medicine and Science, University of Nottingham, Sutton Bonington Campus, Leicestershire LE12 5RD, UK; alaa_tariq2001@yahoo.com (A.T.A.-s.); victoria.marsh@nottingham.ac.uk (V.M.); 2 School of Biosciences, University of Nottingham, Sutton Bonington Campus, Loughborough LE12 5RD, UK; carl.stevenson@nottingham.ac.uk; 3 Biodiscovery Institute, School of Chemistry, University of Nottingham, Nottingham NG7 2RD, UK; huw.williams@nottingham.ac.uk

**Keywords:** *Toxoplasma gondii*, blood–brain barrier, host response, host-pathogen interaction, metabolomics, verapamil

## Abstract

*Toxoplasma gondii* (*T. gondii*), the causative agent of toxoplasmosis, is a frequent cause of brain infection. Despite its known ability to invade the brain, there is still a dire need to better understand the mechanisms by which this parasite interacts with and crosses the blood–brain barrier (BBB). The present study revealed structural and functional changes associated with infection and replication of *T. gondii* within human brain microvascular endothelial cells (BMECs) in vitro. *T. gondii* proliferated within the BMECs and disrupted the integrity of the cerebrovascular barrier through diminishing the cellular viability, disruption of the intercellular junctions and increasing permeability of the BMEC monolayer, as well as altering lipid homeostasis. Proton nuclear magnetic resonance (^1^H NMR)-based metabolomics combined with multivariate data analysis revealed profiles that can be attributed to infection and variations in the amounts of certain metabolites (e.g., amino acids, fatty acids) in the extracts of infected compared to control cells. Notably, treatment with the Ca^2+^ channel blocker verapamil rescued BMEC barrier integrity and restricted intracellular replication of the tachyzoites regardless of the time of treatment application (i.e., prior to infection, early- and late-infection). This study provides new insights into the structural and functional changes that accompany *T. gondii* infection of the BMECs, and sheds light upon the ability of verapamil to inhibit the parasite proliferation and to ameliorate the adverse effects caused by *T. gondii* infection.

## 1. Introduction

*Toxoplasma gondii* is an obligate intracellular apicomplexan protozoan, known to infect at least one-third of the global human population [1,2]. *T. gondii* is also known to invade and proliferate within all eukaryotic cells and infect an extensive number of warm-blooded mammals. During the hematogenous dissemination of *T. gondii*, tachyzoites travel through the blood stream to invade tissues that are anatomically distant from the original site of infection. However, the brain is the most common site where the latent stage of *T. gondii* is normally found [3,4]. Once inside the brain parenchyma, *T. gondii* preferentially infects neuronal cells and develops persistent tissue cysts in the brain [5]. Experimental evidence indicates that *T. gondii* crosses the blood–brain barrier (BBB) using a ‘Trojan horse’ mechanism, breaching the BBB paracellularly by targeting tight junction proteins or crossing the BBB by direct infection of endothelial cells [6,7,8,9].

Foci of *T. gondii* were observed surrounding microvessels in mice during reactivation of latent infection [10] and acute infection [11], indicating that *T. gondii* likely enters the brain through the vasculature, by breaching the formidable BBB [8]. To do this, they must adhere to, and cross, the endothelial cells lining blood vessel walls. In a study in mice, vascular endothelial cells were shown to serve as a replicative niche for the parasite which, after replication and bursting of endothelial cells, infected adjacent brain cells [11]. *T. gondii* infection was found to deregulate radial glia (RG) cell proliferation, differentiation potential, and decrease TGF-β1 levels. *T. gondii* infection of RG cells resulted in impairment of endothelial cell barrier functions through disorganization of cell junction-associated ZO-1 and reduced trans-endothelial electrical resistance (TEER) [12]. Infection of primary human umbilical vein endothelial cells by *T. gondii* caused alteration of the morphology, barrier permeability, and transcriptional profile of the infected cells. *T. gondii* also disrupted the vascular endothelial cadherin and β-catenin localization to the cell margin, reduced vascular endothelial cadherin protein expression, and reduced filamentous actin stress fiber abundance under static and microfluidic shear stress conditions [13].

In a case-control study of patients with acute toxoplasmosis, serological evidence of *T. gondii* infection was associated with significant oxidative stress and pro-inflammatory changes which may contribute to development of endothelial dysfunction [14]. *T. gondii* infection of mice increased vascular inflammation, leukocyte-endothelial cell interaction, vasodilation, and BBB permeability [15]. Using a transgenic mouse model, Portillo et al. [16] showed that during interaction of infected leukocytes with endothelial cells, upregulation of CD40 and induction of autophagy protein-dependent anti-parasitic activity in endothelial cells restricts *T. gondii* invasion of neural tissue. Despite these commendable efforts, the mechanisms by which *T. gondii* subverts the function of the BBB cells and crosses the BBB to cause neuropathy remain largely unresolved.

Elucidating the initial interaction between *T. gondii* and brain vascular endothelial cells is important because this sets the stage for ensuing infection and may determine success, defined by establishment, survival, breach of the BBB, and dissemination to the central nervous system. While the outcomes of *T. gondii* interaction with the BBB reflect the properties of the parasite agent, it is also important to understand the response of the host BBB cells to infection. Although important in neuro-pathogenesis, the interaction of *T. gondii* with brain microvascular endothelial cells (BMECs) has received less attention than other cell types. Therefore, in the present study, we investigated the phenotypic and functional changes that occur in the primary human BMECs during *T. gondii* infection. Our data provide new insights into the molecular mechanisms underpinning *T. gondii*-induced BMEC barrier dysfunction. We also show that the Ca^2+^ channel blocker verapamil significantly reduced the proliferation of *T. gondii* tachyzoites and ameliorated the adverse impact of infection on barrier function.

## 2. Materials and Methods

### 2.1. Maintenance of Cell Cultures

BMECs were grown at 37 °C in a humidified 5% CO_2_ atmosphere, as described previously [17,18]. Cells were cultured in Roswell Park Memorial Institute (RPMI) medium (Gibco, Paisley, UK) supplemented with 20% heat-inactivated fetal bovine serum (FBS); 2 mM L-glutamine; 1% sodium pyruvate; 1% non-essential amino acids; 1% vitamin, and 1% antibiotic-antimycotic solution (10,000 units/mL penicillin, 10,000 µg/mL streptomycin, and 25 µg/mL Amphotericin B). Cells were sub-cultured once a week. On reaching confluence, the cells were treated with trypsin- ethylenediaminetetraacetic acid (EDTA) and sub-cultivated at a ratio of 1:3 in new T-75 cm^2^ NUNC™ culture flasks with fresh RPMI medium. Cell viability was assessed with a hemocytometer following staining with 0.15% trypan blue solution. Media, FBS, media supplements, and antibiotics were purchased from Gibco (Life Technologies, Waltham, MA, USA). Flasks were incubated at 37 °C in a 5% CO_2_ atmosphere, and cultured media were renewed twice a week. Madin-Darby Canine Kidney cells (MDCK) were obtained from the European Collection of Authenticated Cell Cultures (ECACC, Salisbury, UK). MDCK cell cultures were maintained in complete Dulbecco’s modified Eagle’s medium (DMEM; Gibco, Paisley, UK), supplemented with 10% heat-inactivated FBS, 2 mM glutamine, and 1% antibiotic-antimycotic solution at 37 °C in a humidified 5% CO_2_ atmosphere.

### 2.2. Propagation and Purification of Toxoplasma gondii Tachyzoites

Tachyzoites of *T. gondii* RH strain (genotype I) were maintained by passage in MDCK cell cultures. MDCK culture flasks infected with *T. gondii* were observed daily using a Leica DM 1L inverted microscope (Leica Microsystems, Buffalo Grove, IL, USA). When >80% of the cell monolayer were destroyed, a cell scraper was used to detach the remaining cells in the flask. Detached cells and media were collected into a 50 mL tube and centrifuged at 3000× *g* for 5 min. The supernatant was discarded and the pellet was re-suspended in fresh culture medium. Tachyzoites were purified from their feeder-cell cultures by passing through PD-10 desalting columns packed with Sephadex G-25 [19]. The purified parasites were then centrifuged at 800× *g*, re-suspended in fresh culture medium and counted with a Neubauer hemocytometer counting chamber (Marienfeld Superior; Paul Marienfeld GmbH and Co, Lauda-Königshofen, Germany) and a Leica DM 1L inverted microscope.

### 2.3. Infection of Brain Microvascular Endothelial Cells (BMECs)

To optimize the host cell-to-parasite ratio, BMECs were seeded in 24-well hanging cell culture inserts (3.0 µm PET, Merck Millipore, Darmstadt, Germany) at 10^4^ cells/insert. After 5 days of incubation, BMEC monolayers grown in two inserts were trypsinized, and detached cells were counted in order to determine the multiplicity of infection (MOI). The final volume of purified tachyzoite suspension was adjusted with fresh RPMI medium to achieve a MOI of 5 (5 tachyzoites to 1 host cell) in each insert, this was used in all subsequent experiments, unless otherwise stated. Uninfected control cells were sham-infected with an equal volume of RPMI medium devoid of tachyzoites. After 3 h of incubation, the media from all wells were discarded and replaced with fresh RPMI medium. All experiments were performed in triplicate, and the data represent the mean ± standard error of the mean (SEM) from at least three independent experiments.

### 2.4. Phenotypic Characterization of Infection

#### 2.4.1. AlamarBlue^®^ Viability Assay

The effect of *T. gondii* infection on cell viability was assessed with the alamarBlue^®^ assay according to the manufacturer’s instructions (Invitrogen, Carlsbad, CA, USA). This assay is based on the reduction of blue non-fluorescent resazurin (7-Hydroxy-3H-phenoxazin-3-one 10-oxide) to pink fluorescent resorufin, and finally to dihydroresorufin, by the action of cytoplasmic and mitochondrial enzymes in viable cells. AlamarBlue^®^ reagent is an oxidation-reduction indicator that fluoresces red when it accepts electrons generated by cellular metabolism. The quantity of resorufin produced is directly proportional to cellular metabolic activity (i.e., indirectly proportional to the number of living cells). BMECs were seeded in 96-well culture plates, at 5 × 10^3^ cells/well, and grown in 100 μL of RPMI medium per well at 37 °C in a humidified 5% CO_2_ atmosphere. After 24 h of incubation, the tachyzoites were added to the cell monolayers. At 6, 24, or 48 h post-infection (hpi) 10 μL of the alamar dye was introduced into each well. After 4 h of incubation at 37 °C, color changes were determined with a double-wavelength (570 and 630 nm) measurement on a microplate reader (Labtech International Ltd., Ringmer, UK). Cell survival was expressed as a percentage of the absorbance values measured from wells containing uninfected control cells.

#### 2.4.2. Fluorescence Staining

The growth of *T. gondii* tachyzoites within BMECs was monitored using Acridine Orange (AO; 3-N,3-N,6-N,6-N-tetramethylacridine-3,6-diamine) staining. The AO staining assay is based on the permeation of AO into eukaryotic cell and organelle membranes, and emitting green fluorescence when bound to double-stranded DNA, or red fluorescence when bound to single-stranded DNA or RNA. The developing intracellular tachyzoites were identified by their characteristic shape and size dimensions. BMECs were seeded onto cover slips in six-well cell culture plates (Corning, Corning, New York, NY, USA) at a concentration of 10^5^/mL, in 2 mL of RPMI medium, and allowed to adhere for 24 h. Tachyzoites were then added as detailed above. At 6, 24, or 48 hpi the coverslips were removed from the wells, washed twice in 1x phosphate buffered saline (PBS), fixed in 4% paraformaldehyde (PFA) for 30 min, and stained with 10% AO for 10 min at ambient temperature and protected from light. Following staining, coverslips were washed twice in PBS and mounted onto a microscopic glass slide. Fluorescence was observed using a Leica Microsystems DM-5000B epifluorescence microscope equipped with a charge-coupled-device camera (Leica Microsystems, Buffalo Grove, IL, USA). All images were generated using identical exposure times and camera settings. Quantification of the surface area of the AO-stained tachyzoites was performed using ImageJ^®^ software version 15.0 (https://imagej.nih.gov/ij/).

#### 2.4.3. Scanning Electron Microscopy (SEM)

BMECs were grown to confluence on 12-mm glass coverslips (Corning, NY, USA). Tachyzoites were added in RPMI medium and incubated at 37 °C for 6, 24, or 48 hpi. Medium was then removed, and monolayers were rinsed in 1 M cacodylate buffer, fixed with 5% (*w*/*v*) glutaraldehyde in 0.1 M Na-cacodylate buffer for 30 min at ambient temperature, and finally washed with 0.1 M Na-cacodylate buffer. The cells were post-fixed with 1% aqueous osmium tetroxide in 0.1 M Na-cacodylate buffer, pH 7.4, at 25 °C for 90 min, and washed with 0.1 M Na-cacodylate buffer. Following fixation, cells were dehydrated with a 15% graded ethanol series, and dried in a critical point dryer. The samples were then coated with a 20 nm layer of gold/palladium (60:40) in a Balzersunion coater for 15–30 min to increase the specimen’s conductivity. Images were obtained with a Jeol 6060 LV SEM (JEOL, Tokyo, Japan) and using the in-built SEM Control User Interface software (version 6.57).

#### 2.4.4. Transmission Electron Microscopy (TEM)

Tachyzoites were added to confluent monolayers of BMECs grown on Thermo Scientific™ Nunc™ Thermanox™ Coverslips (13 mm in diameter) in 24-well plastic plates in RPMI medium and incubated at 37 °C in 5% CO_2_. At 6, 24, or 48 hpi, coverslips with monolayers were washed three times in 0.1 M cacodylate buffer for 10 min at ambient temperature, and fixed in electron microscopy (EM) fixative buffer (3% glutaraldehyde prepared in 0.1 M cacodylate buffer) for 60 min at 4 °C. After fixation, each specimen was washed three times in the same buffer for 10 min, and post-fixed for 30 min with 1% aqueous osmium tetroxide prepared in 0.1 M cacodylate buffer. Thereafter, each specimen was washed in water five times for 1 min. The specimens were subsequently dehydrated in a graded series of ethanol dilutions using the following protocol: Twice in 50% ethanol for 5 min; twice in 70% ethanol for 5 min; twice in 90% ethanol for 5 min; and thrice in 100% ethanol for 10 min. The specimen was then infiltrated and polymerized with resin, sectioned, and stained with ethanolic uranyl acetate followed by lead acetate. Cells were imaged with a FEI Tecnai G2 12 Biotwin TEM system (FEI, Eindhoven, Netherlands) run at an acceleration voltage of 120 Kilo volt.

### 2.5. Analysis of Neutral Lipids

LipidTox™ is a green neutral lipid stain that detects intracellular lipid droplets (LDs) within mammalian cells. This assay is based on the permeation of LipidTox into cell and tachyzoite membranes and emitting green fluorescence when bound to LDs. BMECs were seeded on six-well cell culture plates on cover slips (Corning, NY, USA) at a density of 10^5^/mL in RPMI medium. Once a confluent cell monolayer is formed (~24 h) tachyzoites were added as described above. The cover slips were removed from the wells at 6, 24, or 48 hpi, washed twice in PBS, fixed in cold acetone:methanol (1:1 *v*/*v*) for 10 min and stained with LipidTox in a 1:400 dilution for 30 min as per the manufacturer’s instructions (Invitrogen, Carlsbad, CA, USA). The nucleus was counter-stained with ProLong Gold antifade reagent with 4′,6-diamino-2-phenylindole dihydrochloride (DAPI; Molecular Probes, Eugene, OR, USA). Samples were observed under a Zeiss LSM880 confocal laser scanning microscope (Carl Zeiss, Oberkochen, Germany) equipped with a monochrome C-4742-95 camera (Hammamatsu Photonics KK, Japan). Quantification of the surface area of stained cells (*n* = 20 cells/group) was performed using image-Pro Plus^®^ software version 6.0 (Media Cybernetics Inc, Rockville, MD, USA).

### 2.6. Endothelial Cell Integrity and Permeability Studies

The influence of infection on the function of the BMEC monolayer was investigated using a TEER assay, AO staining of the BMEC monolayer on the insert mesh, and a fluorescein isothiocyanate-labeled dextran (FITC-dextran) transmigration assay.

#### 2.6.1. TEER Measurements

We measured the electrical resistance across the BMEC monolayer using the TEER assay in order to assess the integrity and permeability of the BMEC monolayer following T. gondii infection. Higher electrical resistance indicates a higher integrity and lower permeability of the cell monolayer, whereas low electrical resistance indicates a compromised, more permeable cell monolayer. Cells were grown on cell culture inserts (0.4 µm pore size) (24-well Millicell^®^ Cell Culture Inserts, Millipore, Watford, UK) fitted in 24-well cell culture plates at 5 × 103 cells/well in RPMI medium, at 37 °C in a humidified 5% CO2 atmosphere. Growth medium was replaced with fresh medium the following day. On day five after culturing, the cell monolayers became confluent and tachyzoites were added. TEER was measured using an EVOM Voltohmmeter and STX2 electrodes (World Precision Instruments, Inc., Sarasota, FL, USA). The electrodes were equilibrated by inserting into a well with the cell culture insert without cells, but with RPMI medium for 5 min at ambient temperature. The value of the blank wells always increases the total resistance measured across a cell culture membrane, and this value was subtracted from the resistance reading of wells with cells to obtain ‘actual resistance.’ The electrodes were used to measure the control wells first, followed by the infected wells. Also, the electrodes were cleaned with 70% ethanol and rinsed with sterile PBS between measurements to avoid cross-contamination of the uninfected culture inserts. One electrode was inserted in the medium covering the BMEC monolayer, while the other electrode was inserted in the medium on the outside of the transwell. TEER readings were recorded at 0, 3, 6, 24, 48, 72, 96, 120, 144, and 168 hpi. To calculate TEER (Ω·cm^2^), electrical resistance reading of inserts without cells was subtracted from the readings obtained from inserts with cells, and this value was multiplied by the surface area of the insert (0.33 cm^2^).

#### 2.6.2. Fluorescein Isothiocyanate (FITC)-Dextran Leakage through the BMEC Barrier

The integrity of the BMEC monolayer grown on Transwell plates was assessed using FITC-dextran (4000 molecular weight, Sigma-Aldrich). Purified tachyzoites were added to confluent cells plated on Transwell plates. The plate was incubated for 1 h at 37 °C and then, 0.3 mL of FITC-dextran in culture medium (1 mg/mL) was added into the upper chamber in the Transwell inserts (the apical side), while 1.3 mL complete RPMI was added into the lower chamber. Monolayer permeability was assayed by measuring FITC-dextran leaked into the bottom chamber of the Transwell (the basolateral side) at 3, 6, 24, 48, and 72 hpi. At each time point after infection the medium in the lower chamber was pipetted several times to evenly mix the leaked FITC-dextran with culture medium before 200 µL of the media was transferred into two wells of 96-well plate, 100 µL/well. Fresh medium (200 µL) was added to each lower compartment to replace the collected medium. The leaked FITC-dextran concentration from the upper chamber to the lower chamber of each well was measured with a Varioskan Flash plate reader (Thermo Fisher Scientific, MA, USA) in ‘Fluorometric Measurement’ mode, with excitation and emission wavelengths of 485 nm and 518 nm, respectively. The leakage of FITC-dextran across the inserts was calculated as a percentage of the total amount added to the upper chamber. The fold change in FITC-dextran fluorescence intensity in infected wells, over that of mock-treated controls (inserts with BMECs and FITC-dextran), was used as a measure of the paracellular permeability of the BMEC monolayers. We also examined the resistance of the BMEC monolayer following *T. gondii* infection, in the presence or absence of FITC-dextran, using the TEER assay. Measurements were recorded up to 168 hpi.

#### 2.6.3. Evaluation of Parasite-Crossing Using Carboxyfluorescein Succinimidyl Ester (CFSE) Staining

The CFSE stain was mixed with *T. gondii* at a concentration of 2 μL CFSE: 200 μL suspension of *T. gondii* tachyzoites. The mixture was incubated at 37 °C in 5% CO_2_ for 3 h, followed by centrifugation for 3 min at 3500× *g*. The supernatant was discarded and the pellet was re-suspended in 1 mL of RPMI medium. The number of stained tachyzoites was counted and added to wells in the Transwell plates. RPMI medium was used to top-up the wells. Five hundred microliters was removed from under the hanging insert at 3, 6, 24, 48, 72, 96, 120, 144, 168, and 192 hpi and the number of tachyzoites was determined with a Neubauer hemocytometer counting chamber (Marienfeld Superior; Paul Marienfeld GmbH and Co, Lauda-Königshofen, Germany) and a Leica DM 1L inverted microscope (Leica Microsystems, Buffalo Grove, IL, USA). An equal volume of fresh RPMI medium was added to the bottom chamber, below the insert, to replenish the medium used for counting the parasites that crossed the BMEC monolayer grown on the insert membrane. We also tested whether the decreased integrity of the BMECs was due to the addition of *T. gondii* only or aggravated by FITC-dextran, by comparing the number of tachyzoites crossing the BMEC monolayer in the presence or absence of FITC-dextran. Measurements were recorded until 168 hpi using a hemocytometer.

#### 2.6.4. Effect of Infection on the Integrity of the BMEC Monolayer

Once the TEER experiment was finished inserts were emptied completely, rinsed gently using distilled water, and fixed in 4% PFA for ~15 min. Inserts were washed again with distilled water before 100 µL of 2% AO was added to control and *T. gondii*-infected BMECs. Inserts were kept in the dark for 20 min, followed by washing with distilled water. The mesh at the bottom of the inserts was removed using a scalpel and was placed on a glass slide. The mesh was imaged using a Leica DM 1L inverted microscope (Leica Microsystems, Buffalo Grove, IL, USA). Also, we used carboxyfluorescein succinimidyl ester (CFSE; Celltrace™ Thermo Fisher, Altrincham, UK) staining to compare the effect of *T. gondii* on the intactness and integrity of the BMEC monolayer. CFSE stain was added to control and *T. gondii*-infected BMECs. The mesh was imaged using a Leica DM 1L inverted microscope and a Leica DM5000 B (Leica Microsystems, Buffalo Grove, IL, USA).

### 2.7. ^1^H NMR-Based Metabolomics

#### 2.7.1. Sample Preparation and Data Acquisition

BMECs were seeded in T75 (75 cm^2^) NUNC™ tissue culture flasks (2 × 10^6^ cells/flask) (Fisher Scientific, Leicestershire, UK) and grown in RPMI medium as described above. Once a confluent cell monolayer was formed (~24 h), the tachyzoites were added at a MOI of 2. At 6, 24, and 48 hpi, the infected and uninfected cells were harvested using a cell scraper, followed by washing three times with cold PBS. The BMECs (infected and uninfected) and tachyzoite pellets were extracted with 2 mL of cold methanol/water (80:20) [20]. Purified tachyzoites were processed separately to identify the metabolic structure of the tachyzoites without any contribution from the host cells. The cells and tachyzoites were subjected to snap-freezing in liquid nitrogen and defrosted on dry ice in three cycles. The cells including the tachyzoites were pelleted by centrifugation at 20,400× *g* for 5 min at −9 °C. The supernatant was transferred to a clean tube and lyophilized. Prior to NMR analysis, the dried extract was reconstituted in 550 μL of deuterated water [D_2_O, 99.9%; Sigma-Aldrich] containing 0.01% of sodium 3-(trimethylsilyl) propionate-2, 2, 3, 3-d4 [TSP, Sigma-Aldrich] as an internal standard. The total volume (550 μL) of the solution was then transferred into 5 mm NMR tubes for subsequent analysis. The data represent at least three independent experiments with six replicates per experiment to identify alterations in the metabolism of infected BMECs compared to uninfected control cells. All ^1^H NMR spectra from the BMECs (infected and uninfected) and the purified tachyzoite extracts were obtained at 600 MHz using a Bruker AVANCE III spectrometer (Bruker Biospin; Rheinstetten, Germany) equipped with 5 mm Broadband/^1^H probe (Bruker SMART probe). Data were acquired using water pre-saturation and 32K data points were acquired and averaged using 512 scans.

#### 2.7.2. Spectra Processing and Multivariate Analysis

NMR spectra were processed with an exponential window function of 0.3 Hz line broadening prior to Fourier transform. Spectra were then individually phased, baseline-corrected, and calibrated to the TSP reference peak at δ 0.00 in Topspin (version 3.1, Bruker). The region containing residual water resonance (4.7 ppm) was excluded to avoid any interference with the data analysis. The spectra were normalized over the total remaining spectral area using MATLAB (R2008a, MathWorks, Inc., 2008, Natick, Massachusetts, US) to improve data quality and remove biases. Multivariate statistical analysis, including principal component analysis (PCA), partial least squares discriminant analysis (PLS-DA), and orthogonal projections to latent structures (OPLS)-DA were performed on the normalized spectra using SIMCA-P+ 11.0 software (Umetrics, Umea, Sweden). First, we used an unsupervised PCA approach to detect intrinsic clusters and possible outliers within the data set. Next, supervised PLS-DA modeling was applied to improve class discrimination between the two groups (infected versus control). PLS-DA utilizes prior information of class membership and hence optimizes separation [21]. The validity of the model was evaluated using *R*^2^ and *Q*^2^ values, where *R*^2^ represents the proportion of variance in the data explained by the model and suggests goodness of fit, and *Q*^2^ shows the predictability of the model. *R*^2^ and *Q*^2^ > 0.5 indicate that the model is fit and robust. Additionally, OPLS-DA was used to maximize the separation between the infected group and the control group. This method enhances the recovery of infection-related metabolites and removes variation unrelated to infection status using an orthogonal signal correction (OSC) filter. To identify the individual metabolites responsible for discriminating infected BMECs from controls, color map coefficient plots were applied.

### 2.8. The Effect of Verapamil on BMEC Function

We investigated the inhibitory effect of verapamil (Sigma-Aldrich; St Louis, MO, USA) on the proliferation of *T. gondii* tachyzoites and on offsetting the increased permeability of the BMEC monolayer caused by *T. gondii* infection. We tested the hypothesis that verapamil could enhance the BMEC monolayer integrity by limiting parasite growth.

#### 2.8.1. Inhibitory Effect of Verapamil on *T. gondii* Growth

We investigated whether pre-treatment with verapamil inhibited the in vitro growth of tachyzoites, by incubating freshly purified tachyzoites with three concentrations of verapamil (1 μM, 10 μM, or 100 μM) for 3 h at 37 °C and 5% CO_2_. The treated tachyzoite suspension was centrifuged at 3000× *g* for 5 min. The supernatant was discarded and the parasite pellet re-suspended in 1 mL RPMI medium. The pre-treated tachyzoite suspension was added at a MOI of 5 to BMECs seeded in 24-well culture plates at ~2 × 10^4^ cells/well in 1 mL RPMI medium, and the wells were topped-up with RPMI to 2 mL per well. We also tested the antiparasitic effect of early verapamil treatment. Each well with cell monolayer in 2 mL RPMI medium was infected with *T. gondii* at a MOI of 5, or mock-infected with medium only. The plate was incubated for 3 h at 37 °C in 5% CO_2_. Media were then removed from the wells and verapamil was added at the three concentrations in RPMI medium. In a third experiment, the antiparasitic effect of late verapamil treatment was also tested. Confluent cells grown in 2 mL RPMI medium in wells were infected with *T. gondii* at a MOI of 5, or mock-infected using medium without tachyzoites. The plate was incubated for 24 h at 27 °C and 5% CO_2_. Medium was then removed from the wells and verapamil was added at the three concentrations in RPMI medium. The plates were checked every 24 h to monitor the tachyzoites’ egress from the cells using a Leica DM 1L inverted microscope (Leica Microsystems). Once the tachyzoites exited the control (untreated) infected cells (~3 days post infection), 1 mL of medium was removed from each well, and the number of extracellular parasites was counted using a Neubauer hemocytometer counting chamber and Leica DM 1L inverted microscope.

#### 2.8.2. Effect of Verapamil on BMEC Monolayer Integrity

BMECs were seeded on microporous polycarbonate membrane filters (3 μm pore size; 24 mm diameter; Transwell 3414; Costar, Corning, NY, USA), and confluent monolayers were infected with tachyzoites at a MOI of 5 or mock-infected. Changes in the TEER values, and thus the integrity of the cell monolayer following *T. gondii* infection up to 120 hpi, with and without treatment with three concentrations of verapamil (1 μM, 10 μM or 100 μM), were measured using the TEER assay as detailed above.

### 2.9. Statistical Analysis

All statistical analyses were conducted using GraphPad Prism version 7.02 (GraphPad^®^ Software Inc., San Diego, CA, USA). Statistical differences in host cell viability, integrity of EC monolayers, and surface area of the stained lipids between control (uninfected) and infected samples at different times after infection were analyzed using two-way analysis of variance (ANOVA). The Bonferroni test was used for post-hoc analysis as appropriate. Changes in the surface area of AO-stained tachyzoites over time were analyzed using one-way ANOVA and Tukey’s test was used for post-hoc analysis. Data are representative of at least three independent experiments and are expressed as means ± standard errors of the means (SEM) where *p* values < 0.05 were considered statistically significant. Significance for all statistical tests is described in the figure legends.

## 3. Results

### 3.1. Effects of T. gondii on the Viability and Structure of BMECs

**Figure 1:**
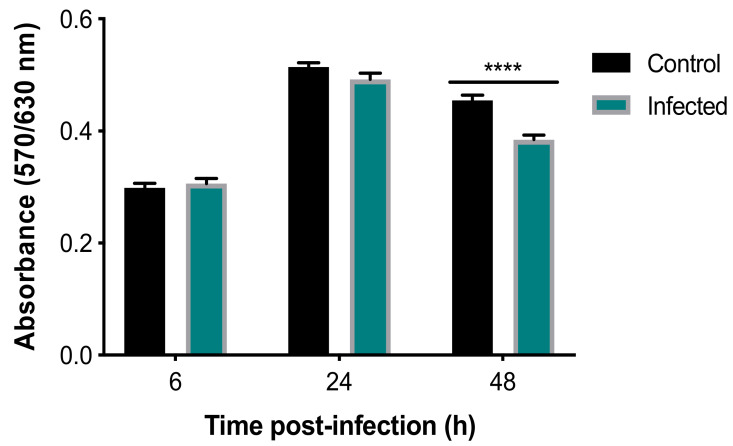
*T. gondii* reduced the viability of BMECs. Absorbance was reduced in infected, compared to control, cells at 48, but not 6 or 24, hpi (****, *p* < 0.0001), indicating reduced viability of infected cells.

**Figure 2:**
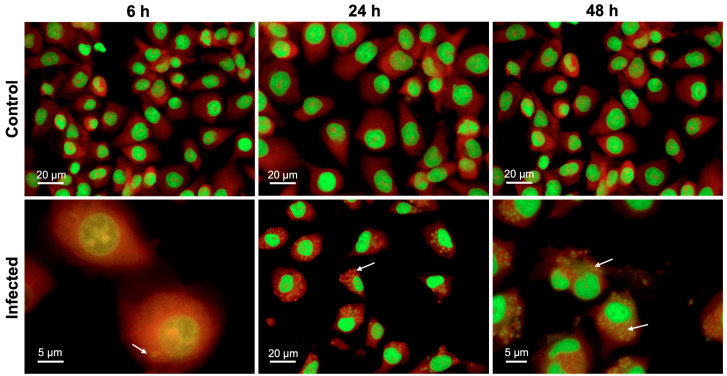
*T. gondii* development within BMECs. Representative images of BMECs infected by *T. gondii* tachyzoites and stained with AO at 6, 24, and 48 hpi. Numerous intracellular tachyzoites (indicated by arrows) were observed in infected BMECs, particularly at 48 hpi. Cellular DNA and RNA are shown in green and red, respectively.

Analysis of the viability of BMECs with an alamarBlue^®^ assay using two-way ANOVA revealed both a significant main effect of group (F (1, 34) = 15.08, *p* = 0.0005) and time (F (2, 68) = 257.3, *p* < 0.0001), as well as a significant group × time interaction (F (2, 68) = 9.697, *p* = 0.0002) (Figure 1). Post-hoc analysis revealed that infected cells showed significantly decreased viability, when compared to uninfected controls at 48 hpi (*p* < 0.0001). As shown in Figure S1, analysis of the AO-stained parasite-specific surface area using one-way ANOVA revealed a significant main effect of time (F (2, 4) = 630.9, *p* < 0.0001) and post-hoc analysis indicated an increase in parasite growth inside infected cells at 48 hpi when compared with 6 and 24 hpi (*p* < 0.001). This result was supported by microscopic examination of stained cells which showed more parasite divisions and larger collections of dividing tachyzoites that were evident at 48 hpi (Figure 2).

**Figure 3:**
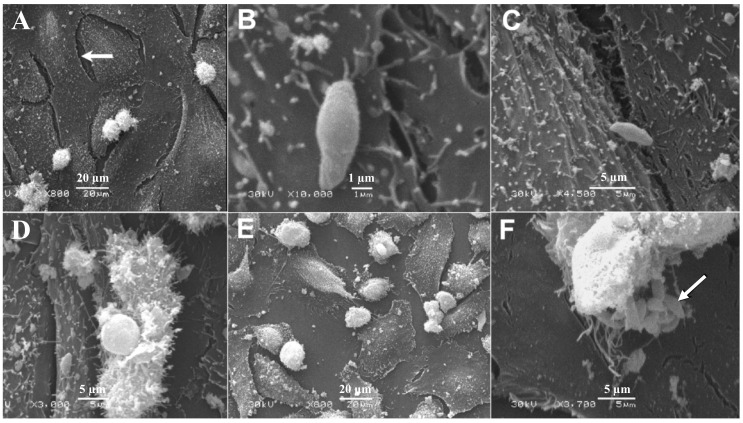
Scanning electron micrographs of BMECs infected by *T. gondii* tachyzoites. (**A**) A confluent monolayer of uninfected control BMECs showing normal intercellular space (arrow). (**B**) At 6 hpi tachyzoites came in close contact with cells, with the anterior ends of the tachyzoites embedded in the intercellular space (**C**). A large gap is evident between infected cells at 24 and 48 hpi (**D**,**E**), compared to 6 hpi (**B**,**C**) and to uninfected control cells (**A**). By 48 hpi tachyzoites were observed exiting from the host cell (**F**) and a number of newly generated tachyzoites were visible (arrow).

**Figure 4:**
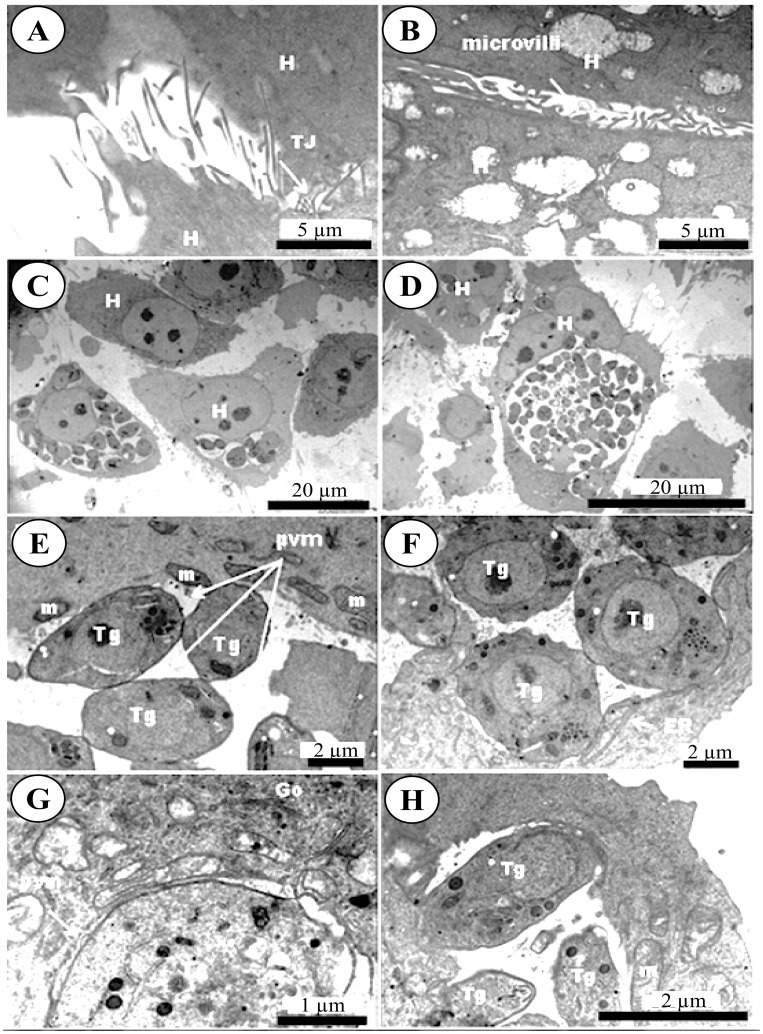
Transmission electron micrographs of BMECs infected by *Toxoplasma gondii* tachyzoites. (**A**,**B**) Control host cell (H) showing microvilli and normal intercellular tight junctions (TJ). Infected cells at 24 hpi (**C**) and 48 hpi (**D**) developed larger gaps as the infection advanced. At 24 hpi mitochondria (m) were found condensed around the parasitophorous vacuole membrane (PVM) that enclosed the tachyzoites (Tg) (**E**). The presence of the ER (**F**) and Golgi apparatus (Go) (**G**) around the PVM was also observed. By 48 hpi tachyzoites were getting ready to exit the PV (**H**).

SEM analysis of BMECs infected by tachyzoites at the early stage of infection (6 hpi), showed crescent-shaped tachyzoites in close contact with host cells (Figure 3B), with their anterior ends embedded in the intercellular space (Figure 3C). Normal tight junctions (TJs) in control cells are shown in Figure 3A and tachyzoites disrupted them over time (Figure 3C–E). By 48 hpi, tachyzoites were observed exiting from host cells (Figure 3F). TEM analysis of uninfected cells showed normal TJs (Figure 4A–B); however, infected BMECs showed that tachyzoite proliferation disrupted the TJs in infected cells over time (Figure 4C–D). Host-cell organelles, including mitochondria (Figure 4E), endoplasmic reticulum (ER) (Figure 4F), and Golgi apparatus (Figure 4G), were observed in close proximity to the parasitophorous vacuole (PV); this became less evident by 48 hpi (Figure 4H).

### 3.2. Perturbed Lipid Homeostasis in Infected Cells

**Figure 5:**
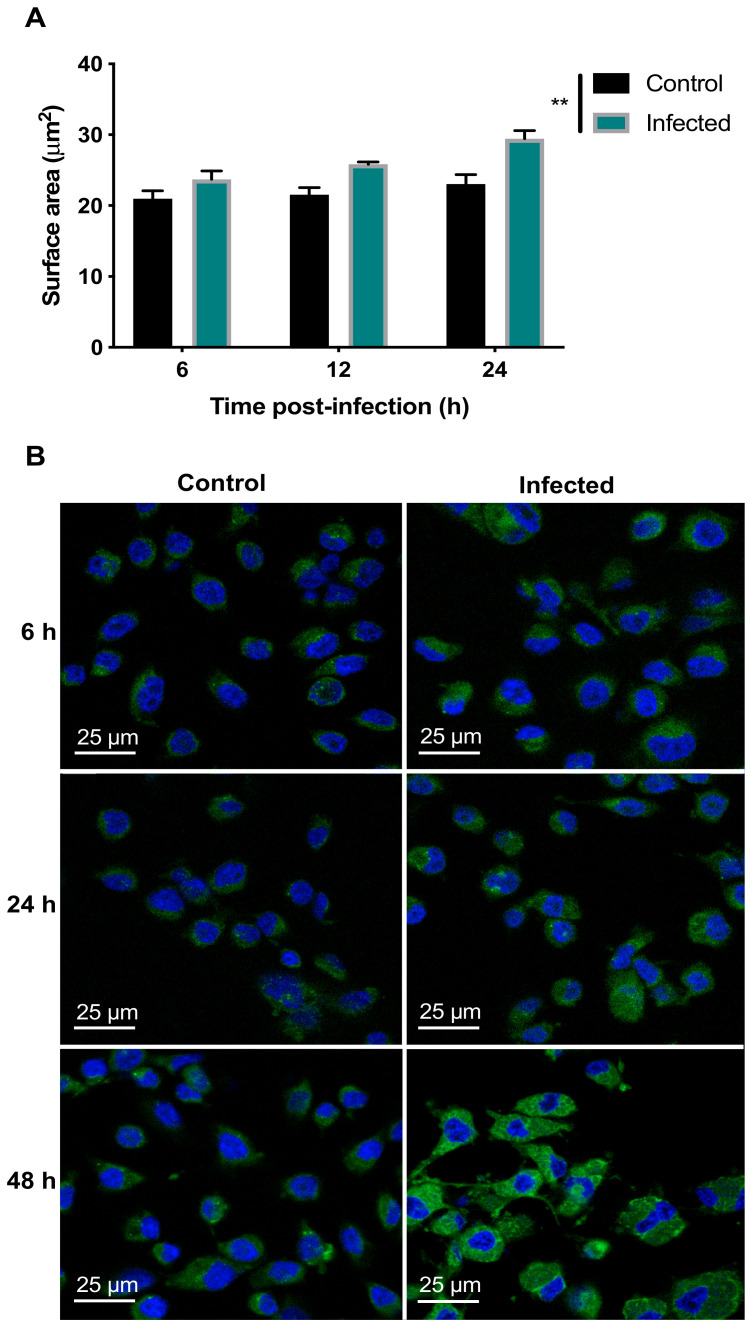
*T. gondii* increased the lipid content of the infected BMECs. (**A**) Infected cells exhibited increased LipidTox™ green fluorescence intensity, compared to control cells (** *p* < 0.01). (**B**) Representative images of BMECs infected by *T. gondii* showing increased lipid staining denoted as green dots and blue host cell nuclei stained with 4′,6-diamidino-2-phenylindole (DAPI).

Analysis of the LipidTox staining using two-way ANOVA revealed significant main effects of group (F (1, 12) = 14.33, *p* = 0.0026) and time (F (2, 24) = 12.7, *p* = 0.0002) but no group × time interaction (F (2, 24) = 2.717, *p* = 0.0864). Post-hoc testing revealed that staining was significantly increased in infected, compared to control, cells across all time points (*p* < 0.01) (Figure 5A). These results are consistent with the microscopic examination of the fluorescent images, which showed slight enhancement in cytoplasmic LD formation in BMECs due to *T. gondii* infection, which was only more prominent at 48 hpi (Figure 5B).

### 3.3. Disruption of BME Barrier Integrity by Infection

**Figure 6:**
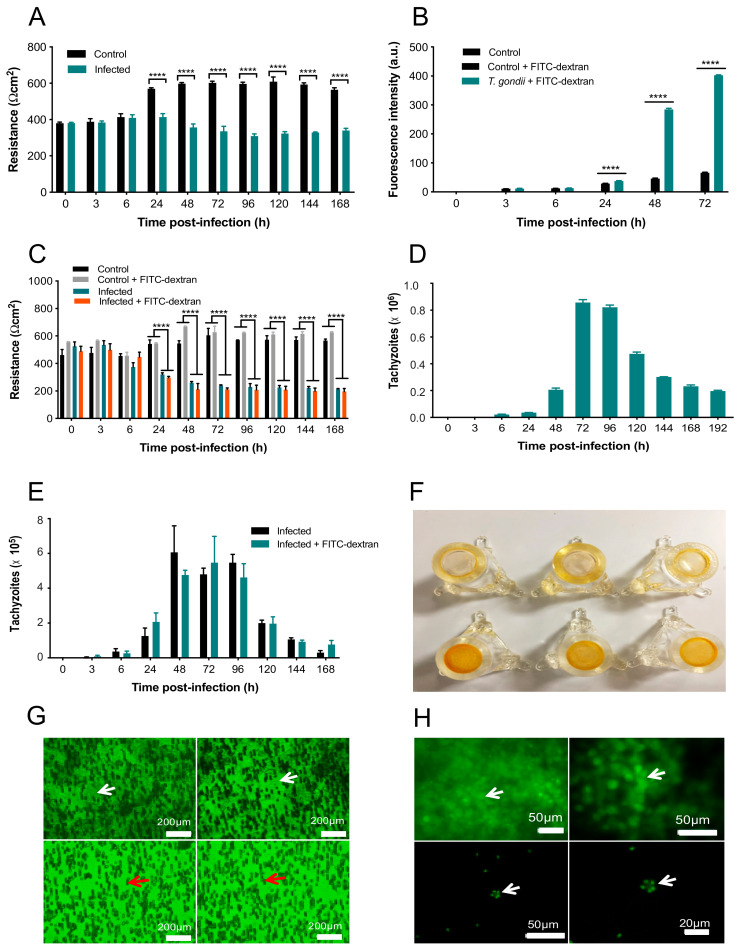
*T. gondii* compromised the BMEC monolayer integrity. (**A**) Infected cells show decreased resistance compared to controls from 24 h onwards. (**B**) *T. gondii* significantly increased FITC-dextran flux in the cell monolayer compared to control BMECs + FITC-dextran. (**C**) Resistance was decreased in infected, compared to control, cells from 24–168 h, regardless of the presence or absence of FITC-dextran. (**D**) There was a large number of tachyzoites crossing the cell monolayer after 48 h. There was still, however, a small number of tachyzoites crossing between 6 and 24 hpi and the numbers started declining after 96 hpi. (**E**) There was no difference between the number of parasites crossing the cell monolayer in infected cells in the presence or absence of FITC-dextran (*p* > 0.05), suggesting the decreased integrity of the cells was due to *T. gondii* only. (**F**) Inserts containing uninfected control cells displayed dark even orange staining (bottom row). Inserts with *T. gondii*-infected cells displayed minimal staining (top row). As AO stain binds to cells, the lack of orange staining indicates few/no cells are present. (**G**) CFSE staining showed that control cells maintained an intact cell monolayer, with arrows indicating notable cell outlines (top row). *T. gondii*-infected cells showed no cells, indicating that the monolayer has been damaged. The dark cylinders are not cells; they are fibers from the insert indicated by red arrows. (**H**) CFSE staining showed an intact control cell monolayer in the top row indicated by green staining (arrow). The bottom row shows a lack of cells; however, tachyzoites were observed (arrow) that were identifiable by the shape and the relative size, indicating that the cell monolayer has been damaged. **** *p* < 0.0001, compared to the control.

To assess the integrity of the BMEC membrane, changes in the resistance of the cell monolayer grown on Transwell inserts following the addition of *T. gondii* were quantified over 168 h using a TEER assay (Figure 6A). The analysis revealed significant main effects of group (F (1, 4) = 206, *p* = 0.0001) and time (F (9, 36) = 20.3, *p* < 0.0001), as well as a significant group x time interaction (F (9, 36) = 52.62, *p* < 0.0001). Post-hoc testing revealed that while the resistance of control and infected cells did not differ from 0–6 hpi (*p* > 0.05), infected cells did show significantly decreased resistance, compared to control cells, from 24–168 hpi (*p* < 0.0001). FITC-dextran was quantified in control and *T. gondii*-infected cells using FITC-dextran leakage analysis (Figure 6B). The analysis revealed significant main effects of group (F (2, 6) = 16,774, *p* < 0.0001) and time (F (5, 30) = 15,150, *p* < 0.0001), as well as a significant group x time interaction (F (10, 30) = 10,349, *p* < 0.0001). Post-hoc testing revealed that while there was no difference in FITC-dextran between control and infected cells from 0–6 hpi (*p* > 0.05), infected cells showed significantly increased FITC-dextran, compared to control cells, from 24–72 hp (*p* < 0.0001). This indicates an increase in barrier permeability due to *T. gondii* infection. 

FITC-dextran was added to *T. gondii*-infected and control cells to determine if differences in TEER were due to *T. gondii* alone or if they also involved FITC-dextran. Adding FITC-dextran to uninfected cells allowed us to determine whether the FITC-dextran caused any further damage to the cell integrity or alter the TEER values (Figure 6C). The analysis revealed significant main effects of group (F (3, 6) = 126.3, *p* < 0.0001) and time (F (9, 54) = 12.45, *p* < 0.0001), as well as a significant group x time interaction (F (27, 54) = 17.35, *p* < 0.0001). Post-hoc testing revealed that there were no differences in resistance between control and infected cells in the presence or absence of FITC-dextran from 0–6 hpi (*p* > 0.05). In contrast, infected cells showed significantly decreased resistance, compared to control cells, from 24–168 h (*p* < 0.0001). There were no differences in resistance between control cells in the presence or absence of FITC-dextran except at 48 hpi, when cells with FITC-dextran showed significantly increased resistance compared to cells without FITC-dextran (*p* < 0.05). Importantly, resistance did not differ between infected cells in the presence or absence of FITC-dextran at any time point examined (*p* > 0.05). This indicates a lack of effect of FITC-dextran on the *T. gondii*-induced increase in BMEC monolayer permeability. 

The number of *T. gondii* tachyzoites crossing the infected cell monolayer was quantified using a hemocytometer at 3, 6, 24, 48, 72, 96, 120, 144, 168, and 192 hpi. As shown in Figure 6D, the first tachyzoites to cross the cell monolayer were observed at 6 hpi, with ~2 × 10^5^ crossing at 48 h; however, at 72 hpi in excess of 8.5 × 10^5^ tachyzoites crossed the barrier. After 96 hpi, the number of tachyzoites crossing the monolayer declined over time. FITC-dextran was also added to *T. gondii*-infected cells to test whether the presence of FITC-dextran would affect the parasite crossing the barrier (Figure 6E). The analysis revealed a significant main effect of time (F (9, 36) = 29.93, *p* < 0.0001) but no main effect of group (F (1, 4) = 0.03489, *p* = 0.8609) or group x time interaction (F (9, 36) = 0.6228, *p* = 0.7697. The majority of tachyzoites crossing the barrier occurred between 48 and 96 hpi but FITC-dextran had no effect.

To visualize the intactness of the BMEC monolayer on the membrane in the inserts, the inserts of infected and uninfected BMEC monolayers were stained with AO. *T. gondii*-infected BMECs showed minimal staining, indicating that few cells remained on the insert membranes. In contrast, control inserts containing BMECs only were evenly stained orange, indicating that the BMEC monolayer remained intact on the insert membranes (Figure 6F). Finally, inserts were stained with CFSE to localize the parasites in relation to the presence or absence of a cell monolayer. Cells can be observed in control samples containing BMECs only (Figure 6G); however, BMECs infected by *T. gondii* had a complete lack of cells, indicating cell loss due to the proliferation of tachyzoites. Control BMECs showed an intact cell monolayer, with arrows indicating notable cell outlines, whereas *T. gondii*-infected BMECs showed no cells present, reflecting the damage in the BMEC monolayer (Figure 6H).

### 3.4. Determination of Metabolic Differences between Infected and Uninfected Cells

**Figure 7:**
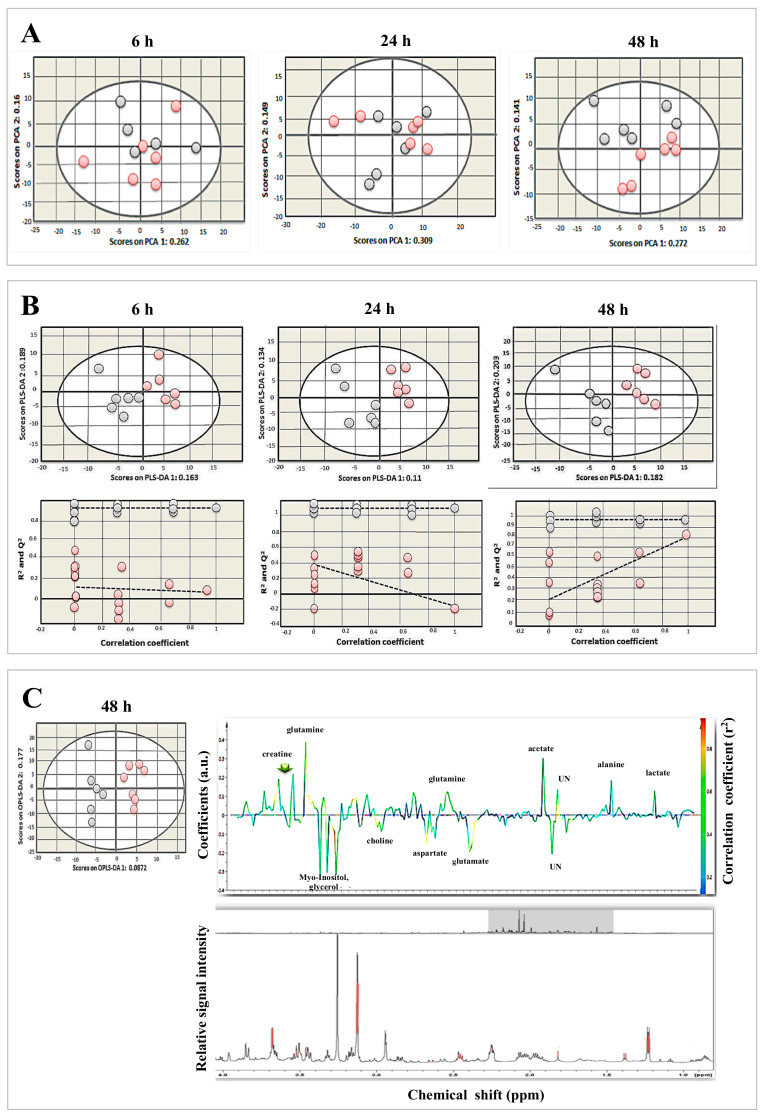
Metabolomic profiling revealed distinct changes in metabolites between *T. gondii*-infected and control cells. (**A**) PCA score plots of metabolites of infected and uninfected BMECs at 6, 24, and 48 hpi. Infected cells were slightly separated from uninfected cells along PC1 (*R*^2^X = 0.262, 0.309, and 0.272 for 6, 24, and 48 hpi, respectively). Each point in the plot represents one replicate ^1^H NMR spectra. Gray and red circles denote control and infected samples, respectively. (**B**) PLS-DA score plots (top row) and cross-validation plots (bottom row) of the metabolites. Infected BMECs were separated from uninfected cells along PLS-DA (*R*^2^X = 0.163, 0.11, and 0.182 at 6, 24, and 48 hpi, respectively). (**C**) OPLS-DA score plots (left) and corresponding coefficient loading plots (right) derived from ^1^H NMR spectra of infected and uninfected cells at 48 hpi. Infected cells were separated from uninfected cells along OPLS-DA 1 (*R^2^*X = 0.0872). The color scaling map on the right-hand side of the coefficient loading plot shows the significance of metabolite variations between infected and uninfected cells. Peaks oriented upward (positive direction) and downward (negative direction) indicate metabolites that are more or less abundant, respectively, in infected cells compared to control cells. The red and blue colors denote increased and decreased levels of metabolites, respectively. The peak height is expressed in arbitrary units (a.u.) relative to the total peak contribution. UN indicates unknown metabolite.

^1^H NMR spectra of infected and uninfected BMECs at different infection times were subjected to multivariate statistical analysis. The dominant metabolites were detected and quantified based on their chemical shifts and signal multiplicity, including isoleucine, leucine, valine, lactate, alanine, acetate, glutamate, glutamine, aspartate, creatine, choline, phosphocholine, glycerophosphocholine, scyllo-inositol, taurine, and myo-inositol (Table S1). To identify the metabolites associated with infection, we employed PCA, PLS-DA, and OPLS-DA. Initially, PCA was applied to the spectra of infected and uninfected BMECs at different infection times. The PCA score plots (two principal components (2 PCs); *R*^2^X = 0.422, 0.458, and 0.413 for 6, 24, and 48 hpi, respectively) showed that spectra of infected cells were separated from those of uninfected cells along PC1 (Figure 7A). To obtain a better discrimination between the spectra of infected and uninfected cells, PLS-DA was applied. The PCA score plots showed minor separations between the indicated groups at 6 and 24 h, but separation became more evident at 48 hpi. As shown in Figure 7B, spectra of infected and uninfected cells were unambiguously divided into two distinctive groups based on their metabolite profile (2PCs; *R*^2^X = 0.352, 0.244, and 0.385 for 6, 24, and 48 hpi, respectively). The cross-validation plots of the PLS-DA model had *R*^2^ Y = 1.662 and Q^2^ = −0.0536 at 6 hpi and *R*^2^Y = 1.782, Q^2^ = −0.31 at 24 hpi. However, cross-validation analysis at 48 hpi showed that *R*^2^Y and Q^2^ intercepts were 1.768 and 0.928, respectively, suggesting goodness of fit and robust predictive power of the model. To identify the main metabolites responsible for discriminating infected from uninfected cells, the OPLS-DA model was applied. As shown in Figure 7C, the OPLS-DA score plots (2 PCs; *R*^2^X = 0.264 for 48 hpi) indicated a separation of the spectra of infected cells from those of uninfected cells along PC1. The color map of the coefficient loading plots revealed significant variations in the abundance of some metabolites between infected and uninfected cells. Lactate, alanine, acetate, glutamine, and creatine were more abundant at 48 hpi in infected compared to uninfected cells. In contrast, a reduction in the level of glutamate, aspartate, choline and myo-inositol, and glycerol was noted in infected compared to uninfected cells. Interestingly, ^1^H NMR profiling of the purified parasites identified, based on the chemical shifts and signal multiplicity, lactate, acetate, glutamate, glycerophosphocholine, scyllo-inositol, taurine, myo-inositol, and glycerol (Figure S2).

### 3.5. Verapamil Inhibits T. gondii Growth and Reduces Barrier Permeability in Infected Cells

**Figure 8:**
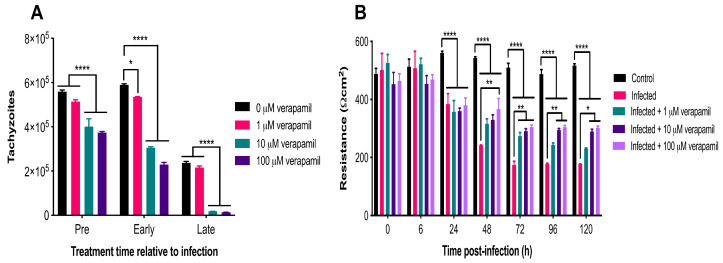
Verapamil inhibited parasite growth and improved barrier function. (**A**) Early treatment with all three concentrations and pre-infection treatment with two concentrations (10 and 100 µM) of verapamil significantly decreased tachyzoite numbers, compared to vehicle control. Late treatment with 10 and 100 µM of verapamil also significantly decreased tachyzoite numbers, compared to vehicle and to 1 µM of verapamil. (**B**) No differences were detected in resistance between control and infected cells treated with any concentration of verapamil from 0–6 hpi (*p* > 0.05). From 24–120 hpi, resistance was significantly reduced in the infected, compared to the control, cells regardless of the verapamil concentration. However, from 48–120 hpi, verapamil resulted in a significant concentration-related increase in resistance in infected cells, compared to untreated infected cells. * *p* <0.05; ** *p* < 0.01; **** *p* < 0.0001.

The antiparasitic activity of three concentrations of verapamil (1 µM, 10 µM and 100 µM) was assessed at three different time points during the parasite infection cycle: (i) Pre-infection treatment (3 h of drug exposure prior to infection), (ii) early treatment (3 hpi), and (iii) late treatment (24 hpi). The number of parasites egressing from cells was counted with a hemocytometer at 72 hpi. The analysis revealed significant main effects of group (F (3, 7) = 314.6, *p* < 0.0001) and time (F (2, 14) = 794.8, *p* < 0.0001), as well as a significant group x time interaction (F (6, 14) = 11.36, *p* = 0.0001). Post-hoc testing revealed that pre-infection treatment with 10 and 100 µM of verapamil significantly decreased tachyzoite numbers, compared to vehicle (*p* < 0.0001). Early treatment with all three concentrations of verapamil also resulted in a significant decrease in the number of tachyzoites, compared to vehicle (*p* < 0.0001). Late treatment with 10 and 100 µM of verapamil also significantly decreased tachyzoite numbers, compared to vehicle and to 1 µM of verapamil (*p* < 0.0001). Interestingly, early treatment with 1 µM of verapamil significantly decreased tachyzoite numbers, compared to vehicle (*p* < 0.05), suggesting the importance of early treatment in controlling the proliferation of the parasite. Overall, verapamil had a significant inhibitory effect on parasite growth at each treatment time point examined (Figure 8A). We also examined the effect of verapamil (1 µM, 10 µM and 100 µM) on the resistance of *T. gondii*-infected compared to control cells up to 120 hpi using the TEER assay (Figure 8B). The analysis revealed significant main effects of group (F (4, 7) = 59.47, *p* < 0.0001) and time (F (6, 42) = 86.66, *p* < 0.0001), as well as a significant group x time interaction (F (24, 42) = 11.93, *p* < 0.0001). Post-hoc testing revealed no differences in resistance between control cells and the infected cells treated with any concentration of verapamil from 0–6 hpi (*p* > 0.05). From 24–120 hpi, resistance was significantly reduced in the infected, compared to the control, cells regardless of the verapamil concentration (*p* < 0.0001). However, from 48–120 hpi, verapamil resulted in a significant concentration-related increase in resistance in infected cells, compared to untreated infected cells (48–96 hpi: *p* < 0.01; 120 hpi: *p* < 0.05). This indicates that verapamil partially mitigated the increase in cell monolayer permeability caused by *T. gondii* infection.

## 4. Discussion

Using BMECs as a simple in vitro BBB model, we showed that *T. gondii* can infect these cells, compromise the intercellular barrier structure and permeability, disrupt cell monolayer resistance, and alter the lipid and metabolomic profiles of infected cells.

### 4.1. Reduced Viability and Altered Structure of Infected Cells

The metabolic activity of BMECs was decreased in *T. gondii*-infected cells 48 hpi. This was probably due to the high energy demand of parasite replication. *T. gondii* alters the metabolism of the host cells or tissues because the parasite exploits host-derived nutrients for its own metabolic processes [22,23,24,25,26]. AO staining enabled the visualization of the progressive development of tachyzoites over 48 hpi. A discernible increase is noted in the surface area corresponding to tachyzoites at 48 hpi. The parasite growth pattern was similar to that observed in human retinal cells [23], macrophages [27], and astrocytoma-derived cells [28] infected by *T. gondii*. Tachyzoites invaded and replicated within cells and, by 48 hpi, several replications had occurred, suggesting that *T. gondii* may enter the human brain by directly interacting with, replicating within, lysing, and breaching the local vascular cells. Previous studies have reported a similar mechanism in polarized epithelial and trophoblastic monolayers [29], in human retinal endothelial cells [30], and in vascular endothelial cells in mice [11]. Ultrastructural analysis revealed damage to cells and diminished integrity of tight junctions. Tachyzoites were observed closely engaged with the cells, and other tachyzoites were exiting from cells after they completed their development. This finding concurs with results observed in Vero cells infected by *T. gondii* tachyzoites [31].

### 4.2. Perturbed Lipid Homeostasis in Infected Cells

*T. gondii* can both synthesize fatty acids (FAs) de novo, via the fatty acid synthase (FAS) I and FAS II systems, and scavenge FAs from its host cells [32]. Intracellular *T. gondii* can acquire selected exogenous fatty acids [33], which are rapidly converted into triacylglycerols and cholesteryl esters [34]. We investigated whether cells modulate their lipid repertoire upon infection. Staining with the fluorescent dye LipidTox™ revealed an increase in the formation of LDs in *T. gondii*-infected cells. More LDs were detected adjacent to the parasite and around the PV. The recruitment of lipids by *T. gondii* may be part of a survival strategy by the parasite to surmount a deficiency of cholesterol and other lipids [22,35]. An increase in the content of LDs was also reported in human foreskin fibroblasts [36] and skeletal muscle cells [37], where *T. gondii* infection increased the biogenesis and recruitment of LDs in a time course-dependent manner to the PVM.

### 4.3. Compromised Barrier Integrity and Increased Parasite Crossing

We found that invasion of cells by *T. gondii* adversely affects the integrity of the BMEC monolayer, confirming results reported in GFP^+^ tachyzoites infecting rat endothelial cells [9]. This was supported by disruption in the intercellular barrier formation observed at 24 and 48 hpi using SEM and TEM. Wider intercellular gaps were also observed in retinal pigment epithelium (ARPE-19) cells infected by *T. gondii* [38]. In further support of our findings, *T. gondii* was found to decrease resistance of colorectal adenocarcinoma-derived Caco-2 cells and impair the integrity of the intestinal mucosa via disruption of TJ proteins, including a decrease in occludin and ZO-1 expression, and redistribution of claudin-1, occludin, and ZO-1 in the cytoplasm of intestinal epithelial cells [39].

We also examined the migration of *T. gondii* across the cell monolayer and found that a small number of tachyzoites crossed the monolayer between 6–24 hpi, which may imply a paracellular crossing method because by 24 h the tachyzoites have not exited the host cells yet. The dramatic increase in the number of tachyzoites crossing the cell monolayer after 48 h correlates with the increased FITC-dextran leakage. Thus, it can be assumed that ~48 h is the time at which tachyzoites start to exit and damage the cells, resulting in increased permeability, number of tachyzoites crossing, and FITC-dextran leakage. The increased cell monolayer permeability observed in the present study may be a direct effect of tachyzoite invasion or induced indirectly via immune and inflammatory responses elicited against *T. gondii* infection. Disruption of TJs can be induced by inflammatory mediators and free radicals in response to infection [40,41,42]. Cell rupture that accompanies *T. gondii* egress has been suggested as an explanation for cell damage, causing an overwhelming increase in the number of tachyzoites and increased permeability [43]. Therefore, the decrease in the number of tachyzoites crossing the insert membrane after 96 h could be due to the cells being completely destroyed by that time, resulting in few parasites actually left in the inserts to cross over. Indeed, significant damage and loss of integrity of the infected cell monolayer was observed on the insert membrane stained with AO and CFSE.

### 4.4. Infection-Induced Metabolic Alterations

^1^H NMR analysis was used to study the metabolic profiles of BMECs infected by *T. gondii*. Lactate, alanine, acetate, glutamine, and creatine were elevated at 48 hpi in infected cells. In contrast, a reduction of glutamate, aspartate, choline, myo-inositol, and glycerol was noted in infected cells. Alterations of these metabolites are expected because survival and replication of *T. gondii* depends on the ability of the parasite to acquire most of its needed macromolecules from the host cell [24,25,26]. This highly exploitative relationship induces metabolic pressure on host cells in order to cope with the nutritional needs of the growing parasites [23,24]. *T. gondii* tachyzoites rely on glucose as a carbon source to meet their bioenergy demands for growth [44,45]. Glucose is converted to lactate, alanine, and acetate via glycolysis [46]. An enhanced rate of glycolysis caused an increase in the production of pyruvate, which can be transaminated to lactate via anaerobic glycolysis and to alanine via aerobic glycolysis. The increased glycolytic activity may provide an alternative source of energy when oxidative phosphorylation is compromised by the impaired mitochondrial function that occurs during *T. gondii* infection [47]. Similar results were observed in erythrocytes infected by *Plasmodium berghei* [48,49]. Alanine can also be synthesized from branched chain amino acids, such as valine, leucine, and isoleucine. The acetate level was also increased in infected cells, which has also been observed in erythrocytes infected by *P. berghei* [50]. The increase of acetate in infected cells may be attributed to the biosynthesis activity of *T. gondii* tachyzoites. A previous study using ^1^H-NMR showed that lactic acid and acetic acid are the major end-products of glucose metabolism in *T. gondii* tachyzoites isolated from peritoneal exudate of experimentally infected mice [51]. Our ^1^H-NMR spectroscopic profiling of in vitro-grown tachyzoites showed a similar result, providing evidence for the crucial role of lactic acid and acetic acid in the metabolism of *T. gondii*.

*T. gondii*-infected cells also exhibited an elevated level of glutamine and a reduction of glutamate at 48 hpi. *T. gondii* relies on glutamine as a carbon source to meet its energy demands during the scarcity of glucose [52,53,54]. Glutamine is converted to glutamate and utilized by the mitochondria cellular respiration cycle as either α-ketoglutarate or to succinate via the γ-aminobutyric acid (GABA) shunt. Alteration in the level of glutamine was also detected in mice infected by *T. gondii* [44], in mice infected with *Schistosoma mansoni* [55], and in primary human foreskin fibroblasts infected by Vaccinia virus [56]. The level of aspartate was also decreased in BMECs. The enzyme aspartate transaminase can convert glutamate and oxaloacetate to aspartate and 2-oxoglutarate, depending on the availability of amino acids and cellular respiration cycle intermediates. The level of choline was decreased in infected BMECs, probably caused via uptake by *T. gondii* from the host [57]. Although *T. gondii* can synthesize certain phospholipids, it acquires choline from host cells to ensure enough synthesis of phosphatidylcholine, which is necessary for its replication. Phosphatidylcholine is the most abundant phospholipid in *T. gondii*, accounting for ~75% of the total phospholipids [57]. Altered choline was also observed in human foreskin fibroblasts infected by *T. gondii* [22,57] and in mice infected by *Schistosoma mansoni* [55]. The level of myo-inositol was decreased in infected BMECs. Myo-inositol is a building block for all inositol-containing phospholipids in pathogens. Inositol is converted to phosphatidylinositol in the Golgi apparatus by phosphatidyl-inositol synthase [58]. Reduced inositol was also detected in mice infected by *Schistosoma japonicum* [59]. In our study, an elevated creatine level at 48 hpi may indicate a process of the host response to the infection. Creatine is converted to phosphocreatine by mitochondrial creatine phosphokinase (CPK) [60,61]. Increased CPK in the serum has been previously associated with toxoplasmosis [62] and malaria [63].

### 4.5. Verapamil Ameliorated the Impact of T. gondii on Barrier Integrity

Verapamil treatment seemed to significantly offset the adverse effect of infection on the cell monolayer permeability from 48 h after treatment onwards in a dose-dependent manner. In a recent study, verapamil was shown to inhibit *T. gondii* crossing of BMECs from 1 µM concentration [64]. Similarly, verapamil caused a significant inhibition of the proliferation of *T. gondii* tachyzoites in a dose-dependent way. The antiparasitic effect was more prominent when the drug was applied 24 hpi compared to treatment at 3 hpi or prior to infection. These results confirmed our hypothesis that verapamil, by limiting parasite growth, could enhance cell monolayer integrity and showed that BMECs can be a potential target of this calcium channel-blocking compound, providing a simple BBB model for future testing to modulate endothelial barrier permeability and/or limit brain infection. Whether the enhancement in cell monolayer integrity associated with verapamil treatment is attributed to its direct antiparasitic effect or to a modulatory effect on the P-gp transporter or other biological functions in the BMECs remains to be investigated. Also, it remains to be confirmed whether the ameliorative effect of verapamil observed in vitro is reflective of the in vivo setting.

## 5. Conclusions

This study showed the ability of *T. gondii* to invade, grow, and replicate within BMECs, and to disrupt their viability, lipid synthesis, and structural integrity, as well as induce BMEC barrier dysfunction. This involved reorganization of the host cell organelles around the PV to support parasite growth and replication. In addition, *T. gondii* induced changes in lipid homeostasis in infected cells, presumably to facilitate its multiplication. *T. gondii* also increased BMEC permeability and disrupted the cell monolayer integrity. Differences were detected in the metabolic profile of infected cells. *T. gondii* affected mainly amino acid and fatty acid metabolism of the host cells. Our data also showed the potential of ^1^H NMR coupled with multivariate statistical analysis to identify infection-related metabolomic signatures that can be used in future studies to predict the progression of the toxoplasmosis disease process. Our results also showed that verapamil inhibited *T. gondii* proliferation and countered the disrupting effect of infection on cell integrity and permeability, limiting transmigration of tachyzoites through the cell monolayers. Understanding the mechanisms that regulate these processes, whether mediated by host-derived factors secreted in response to infection or attributed to effector molecules secreted by *T. gondii*, remains to be fully elucidated.

## Supplementary Materials

The following are available online at https://www.mdpi.com/2076-2607/8/9/1386/s1, Figure S1: AO staining of infected BMECs over 48 hpi. A significant increase was observed in the AO-stained surface area over time (6 vs 48 hpi [**** *p* < 0.0001]; 24 vs 48 hpi [*** *p* < 0.001]), Figure S2: Representative ^1^H NMR spectra of purified *Toxoplasma gondii* tachyzoites, Table S1: Metabolites and their ^1^H chemical shifts identified in the extracts of *T. gondii*-infected compared to uninfected control BMECs.
