# Supplementary material for: Structural, Functional, and Metabolic Alterations in Human Cerebrovascular Endothelial Cells during Toxoplasma gondii Infection and Amelioration by Verapamil In Vitro
Source: Microorganisms. 2020 Sep 10;8(9):1386. doi: 10.3390/microorganisms8091386 (PMC7564162; doi:10.3390/microorganisms8091386)
Supplement: Supplementary file 1 [file microorganisms-08-01386-s001.zip › Supplementary files.docx]

**Figure S1.**

**
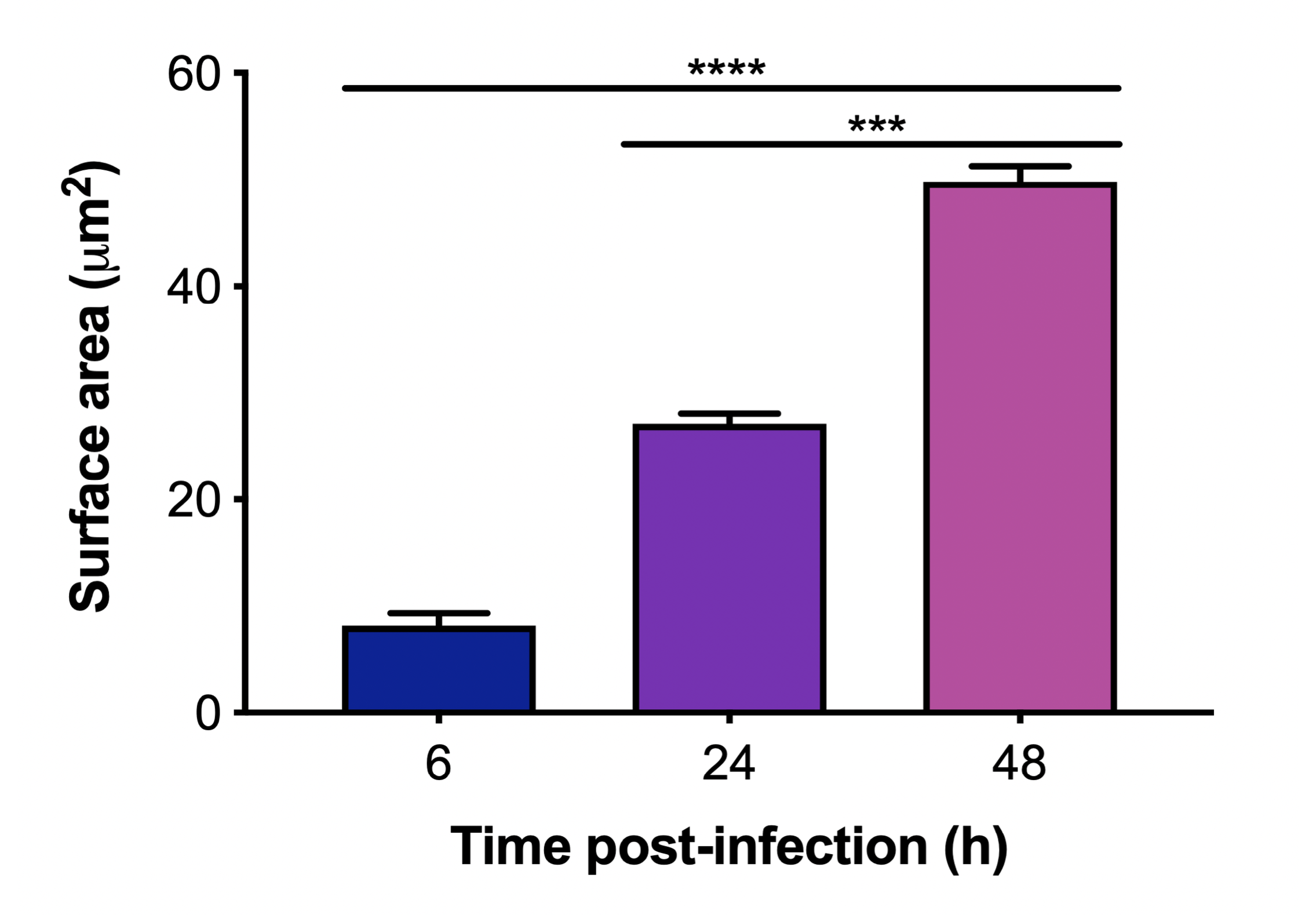
**

**Figure S2.**

**Table S1.** Metabolites and their ^1^H chemical shifts identified in the extracts of T. gondii-infected compared to uninfected BMECs.

| **Metabolite** | **Chemical**  **moiety** | **Signal**  **Multiplicity*** | **Chemical shift(s) in D_2_O [ppm]** | **Main functions** |
| --- | --- | --- | --- | --- |
| Isoleucine | CH  CH  CH_2_  CH_2_  CH_3_  CH_3_ | m  d  m  m  d  t | 1.9692  3.6549  1.4589  1.2492  1.0152  0.9430 | Amino acid:   - protein synthesis - propionic fatty acid precursor |
| Lactate | CH  CH_3_ | m  d  m  m  d  t | 1.9692  3.6549  1.4589  1.2492  1.0152  0.9430 | End-product of anaerobic glycolysis:   - neuronal energy generation - macrophage activation - nitric oxide production - precursor of glutamate |
| Leucine | CH  CH_2_  CH_2_  CH  CH_3_  CH_3_ | qa  d | 4.1140  1.3313 | Amino acid:   - protein synthesis |
| Myo -inositol | CH  CH  CH  CH  CH  CH | m  m  m  t  t  t | 1.6977  1.6740  1.7279  3.7204  0.9591  0.9708 | A cyclic sugar alcohol:   - Osmoregulator - storage form of glucose - central secondary messenger system - hormonal stimulation - precursor of membrane constituents - glial marker |
| N– acetylaspartate | CH  CH_2_  CH_2_  CH_3_ | dd  dd  dd  s | 4.3969  2.6991  2.5062  2.0223 | Amino acid:   - Precursor of acetate - Precursor of aspartate - Precursor of N-acetylaspartate glutamate - ion balance - osmosis in neurons - energy metabolism - neuronal marker |
| Phosphocholine | CH_2_  (CH_3_)_3_ | m  s | 3.5743  3.2264 | An intermediate in the synthesis of [phosphatidylcholine](https://en.wikipedia.org/wiki/Phosphatidylcholine)   - cell membrane constituent |
| Scyllo –inositol | CH | s | 3.3510 | a cyclic sugar alcohol:   - modulates protein folding |
| Taurine | CH_2_  CH_2_ | t  t | 3.2689  3.4312 | An [organic acid](https://en.wikipedia.org/wiki/Organic_acid):   - blockage of N-methyl-D-aspartate receptor - free radical scavenger - calcium homoeostasis - astrocytic osmoregulator - immune system activity - inhibitor neuromodulator |
| Valine | CH  CH  CH_3_  CH_3_ | m  d  d  d | 2.2620  3.5930  1.0464  0.9952 | Amino acid:   - protein synthesis - propionic fatty acid pre-cursor |
| Gamma-amino butyrate | CH_2_  CH_2_  CH_2_ | qi  t  t | 1.9077  2.3025  3.0164 | Amino acid:   - inhibitory neurotransmitter |

* s, singlet; d, doublet; dd, double doublet; t, triplet; qa, quartet; qi, quintet; m, multiplet.
